# Fluorescence Spectroscopy and ^13^C NMR Spectroscopy Characteristics of HA in Black Soil at Different Corn Straw Returning Modes

**DOI:** 10.1155/2021/9940116

**Published:** 2021-06-07

**Authors:** Shuang Zheng, Sen Dou, HongMei Duan, BoYan Zhang, Yue Bai

**Affiliations:** College of Resource and Environmental Science, Jilin Agricultural University, Changchun 130118, Jilin Province, China

## Abstract

A three-year field experiment was conducted to analyze the effects of straw enrichment and deep incorporation on the humus composition and the structure of humic acid (HA) in black soil. The differences in the HA structure between different straw returning methods were detected by three-dimensional fluorescence spectroscopy and ^13^C NMR technology. The purpose of this paper is to provide a theoretical basis and data support for improving the straw returning system. Four different treatments, including no straw applied (CK), straw mulching (SCR), straw deep ploughing (MBR), and straw enrichment and deep incorporation (SEDI: harvested the corn straw from four rows together with a finger-plate rake and then crushed and buried them in one row in the 20∼40 cm deep level in the subsoil with a wind-driven input cylindrical plough), were used in this study. Our results showed that compared to CK treatment, SEDI significantly increased the contents of organic carbon (SOC), soil humic acid carbon (HAC), fulvic acid carbon (FAC), and humin C content (HM-C) in the subsurface soil layer by 27.47%, 34.33%, 19.66%, and 31.49%, respectively. Among all the straw returning treatments, SEDI treatment had the most significant effect in increasing the contents of HEC, HAC, and FAC. Straw returning not only reduced the degree of condensation and oxidation of the HA structure but also increased the proportion of alkyl C and enhanced the hydrophobicity of the HA structure in subsurface soil. Moreover, SEDI treatment significantly increased the proportion of aliphatic C/aromatic C of the HA structure in subsurface soil and improved the aliphatic property of HA, which had a significant effect on the HA structure compared to other treatments.

## 1. Introduction

In recent years, due to long-term intensive agricultural production and soil erosion, the soil organic content and humus quality of black soil have decreased significantly in Northeast China, resulting in shallower soil layers [1] and poor cultivability of land. Moreover, corn straw incorporation was effective in increasing soil organic carbon [2] and humus C content [3], deepening the degree of soil humification [4], and improving the HA structure. Chen et al. showed that straw returning increases the aliphatic group of HA molecular structure, reduces the degree of condensation and oxidation of HA structure, and makes HA structure more lipidic and simple [5].

Soil organic carbon (SOC) pool is the largest terrestrial carbon pool, and 70–80% of SOC is composed of humus [6]. Humic acid (HA) is the main component of humus [7]. Furthermore, the composition, structure, and properties of HA were related to the fertilization characteristics of the soil. Returning straw to the field can improve soil fertility and increase the content of soil organic matter. Different straw returning methods have different effects on the composition of soil humus and the structure of HA. Studies have shown that straw mulching is beneficial to the accumulation of organic carbon and humic substance C content [8] and the enhancement of functional groups such as aliphatic, hydroxyl, methoxy, and carboxyl groups on the surface soil [9], but straw mulching also affects seedling emergence and makes tillage difficult [10]. A new straw returning method had been developed, namely, straw enrichment and deep incorporation (SEDI), consisting in (1) raking the corn stalk in the field together into rows at a ratio of 4 : 1 with a finger-plate rake; (2) crushing the corn straw and burying it into subsoil, 20∼40 cm deep along designated strips with a wind-driven input cylindrical plough; (3) sowing seeds into the strips with no straw buried in between the strips with straw buried in a normal way with a nontillage seeder, to realize separation of the seeded strips (narrow rows) from the strips (wide rows) with straw buried in a wide-and-narrow row alternating cultivation mode. Zuber et al. showed that straw deep ploughing has a better effect on the accumulation of soil organic matter than that of straw mulching [11] and also increases the organic carbon content of HE and HA [12]. Straw deep incorporation can also significantly increase the content of soil subsurface active organic carbon [13], deepen the degree of soil humification [14], reduce the degree of HA structure condensation and oxidation, and increase the content of aliphatic chain hydrocarbons and aromatic carbon. Zhang et al. have shown that straw deep incorporation accumulates soil organic carbon and humus component carbon content in different soil layers and improved the aromaticity and hydrophobicity of HA molecules [15]. Al et al. compared the changes in the structure of soil humic acid after applying crop straw, jujube leaf, and animal manure by solid-state-^13^C NMR. Their results indicated that crop straw has the highest aliphatic content and the strongest aliphatic content [16]. Chen Xi et al. showed that straw returning could increase the relative content of alkoxy carbon in soil organic carbon through ^13^C NMR research [17].

Nowadays, three-dimensional fluorescence spectroscopy and ^13^C NMR techniques were used to study the effects of straw returning on humic composition and HA structure. Previous research mainly focused on straw mulching and straw shallow application on the accumulation of soil organic carbon and humus C in the surface layer [8]. However, the effects of straw enrichment and deep incorporation (SEDI) on soil humus composition and HA structure are yet poorly understood, along with the differences of HA structure between SEDI and other returning methods. To address the issue, we conducted a 3-year field experiment to (i) determine the SOC content, (ii) characterize humus composition and C content, and (iii) compare the changes of HA structure from different return methods by three-dimensional fluorescence spectroscopy and ^13^C CPMAS NMR technology. In order to provide a theoretical basis and data support for the improvement of straw returning system, this paper focused on discussing the impact of the hydrophilicity and hydrophobicity of the HA structure on soil stability.

## 2. Materials and Methods

### 2.1. Experimental Site

The experiment was located at the Experimental Station of Agricultural Technology Extension Center in Jiutai District, Changchun City, Jilin Province (44°08′N, 125°50′E). The experimental soil was classified as Argiudolls. The basic properties of the sampled soil are shown in Table 1.

### 2.2. Experimental Design

The experiment adopted wide-and-narrow row planting. The ridge height was 12 cm, and the row spacing was 40 cm. The planting density was 65000/hm^2^. Four treatments were set randomly with three replications. CK: no straw was applied; SCR: corn straw was evenly placed on the soil surface; MBR: corn straw was evenly placed on the soil surface, and straw deep turning and returning to the field to 25–30 cm soil layer; SEDI: the corn straw was harvested from four rows together with a finger-plate rake and then crushed and buried them in one row in the 20∼40 cm deep level in the subsoil with a wind-driven input cylindrical plough. The corn straw was mechanically crushed in all three corn straw treatments. The experiment selected field planting; each treatment area was 1334 m^2^. Each plot had the same fertilizer application rate: 200 kg·N·ha^−1^, 100 kg·k·ha^−1^, and 100 kg·Pha^−1^.

### 2.3. Soil Sampling and Analysis

Soil samples were collected after the maize harvest in November 2019. For each treatment, the soil samples were collected from three points in each plot replicate. The sampling depth was 0–20 cm and 20–40 cm. Each soil sample was air-dried and passed through a 2 mm sieve to remove plant residues for extracting soil HA.

### 2.4. Laboratory Analysis

SOC was determined using the K_2_Cr_2_O_7_ external heating method. Alkalytic N was measured using the alkali diffusion method; available P was measured using sodium bicarbonate-molybdenum antimony colorimetry; available K was measured using a flame photometer, which was measured by a pH meter [18].

Humus composition was analyzed following the International Humic Substances Society method [19]. Briefly, 5 g of soil sample was sequentially extracted with 30 mL of distilled water, then with 30 mL of 0.1 N mixed alkali solution (NaOH + Na_4_P_2_O_7_) under continuous shaking at 70 °C for one hour. The supernatant was humic extractable (HE) substance. 30 mL HE was acidified to pH 1 to separate HA from humic fulvic (FA). The precipitation was retained as HA and FA remained in the solution. HA was redissolved with 0.05 mol·L^−1^NaOH.

HA isolation and purification were processed using the procedure described by the International Humic Substances Society procedure described by Kuwatsuka et al. [20]. Briefly, 50 g of soil sample was decalcified with HCl; then, residues were extracted by NaOH solution and allowed to stand overnight. The supernatant was soaked in a mixture of HF and HCL solution to remove ash, dialysed to electrodialysis, and freeze-dried afterwards.

### 2.5. Characterization of HA

HA element composition, such as C, H, N, and O, was determined by a Vario EL III elemental analyzer. The fluorescence spectra were obtained at a concentration of 100 mg L^−1^ (pH was adjusted to 8.0 with 0.05 M NaOH). The EEM spectra were recorded with emission wavelength between 300 and 600 nm, while the excitation wavelength was increased sequentially from 250 to 550 nm. The emission-excitation slit was fixed at the 5 nm bandwidth and the scanning speed was set at 12,000 nm min^−1^. The solid-state ^13^C NMR spectra of soil samples were obtained on an AVANCE III 400 WB spectrometer at 100.6 MHz with a spinning rate of 8 kHz, an acquisition time of 34 ms, a recycle time of 5 s, and a contact time of 2 ms. Chemical shift values were externally referenced to the methylene resonance of the adamantane standard at 38.4 ppm. Semiquantification was performed by integration using Mestre Nova 14.0 software [21].

### 2.6. Statistical Analysis

Analysis of variance was performed using SPSS 21.0 software. Statistical significance differences among corn straw returning mode means were evaluated using the least significant difference test at a level of *p* < 0.05.

## 3. Results

### 3.1. SOC and Humic C

**Figure 1:**
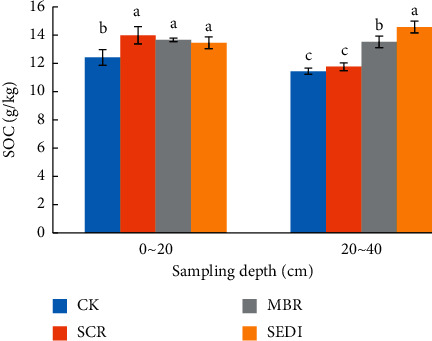
Effect of different treatments of corn straw on SOC.

Data on the C contents of soil for each treatment are presented in Figure 1. Compared with CK treatment, SCR, MBR, and SEDI treatments increased SOC content in the surface soil layer by 12.65%, 10.07%, and 8.38%, respectively. In the 20–40 cm soil depth, the SOC contents increased in the following trend: SEDI > MBR > SCR > CK. All corn straw treatments increased SOC content in the subsurface soil layer by 2.89%, 18.29%, and 27.47%. Thus, it could be seen that for the different straw returning treatments, the SEDI had a more significant cumulative effect on the subsurface organic carbon content.

Data on the humic fractions for each treatment are presented in Table 2. For the humic fractions, SEDI treatment significantly increased the content of humic fraction (HEC) in the 20–40 cm, soil humic acid carbon (HAC), fulvic acid carbon (FAC), and humin C content (HM-C) by 34.33%, 19.66%, and 31.49%, respectively, compared with CK. PQ value is the proportion of HA in humic substances, which can reflect the degree of humification of SOC. Compared with CK, the treatment of straw returning to the field increased the PQ value of the soil to varying degrees. The SEDI treatment increased the PQ value of subsurface soil the most, which increased from 62.71% to 65.39% compared with CK.

### 3.2. Elemental Composition of HA

It can be seen from Table 3 that the elements of HA are mainly composed of C and O, in which the C content ranges from 482.1 g kg^−1^ to 512.9 g kg^−1^, and the O content ranges from 422.5 g kg^−1^ to 479 g kg^−1^. After corn straw returning, the content of C, H, and N in HA increased, while the content of O decreased. Among all the treatments, the SEDI treatment significantly increased the C, H, and N contents in the 20–40 cm depth. In the topsoil, the H/C ratio increased in the trend of SCR > MBR > SEDI > CK, while the O/C ratio decreased in the trend of CK > SEDI > MBR > SCR. In the subsoil, the H/C ratio increased in the trend of SEDI > MBR > SCR > CK, while the O/C ratio decreased in the trend of CK > SCR > MBR > SEDI.

### 3.3. Three-Dimensional Fluorescence EEM Spectra of HA

**Figure 2:**
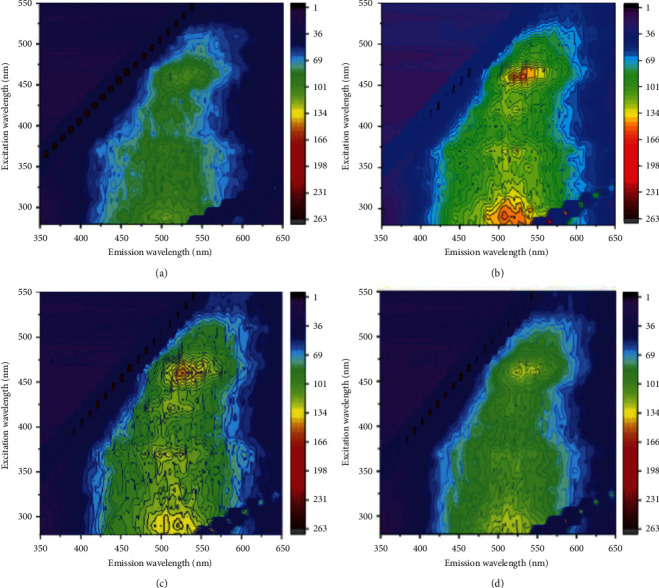
Fluorescence spectra of HA in different maize returning methods on topsoil. *Note.* CK: no straw application; SCR: straw mulching; MBR: straw deep ploughing; SEDI: straw enrichment and deep incorporation. (a) Topsoil: CK. (b) Topsoil: SCR. (c) Topsoil: MBR. (d) Topsoil: SEDI.

**Figure 3:**
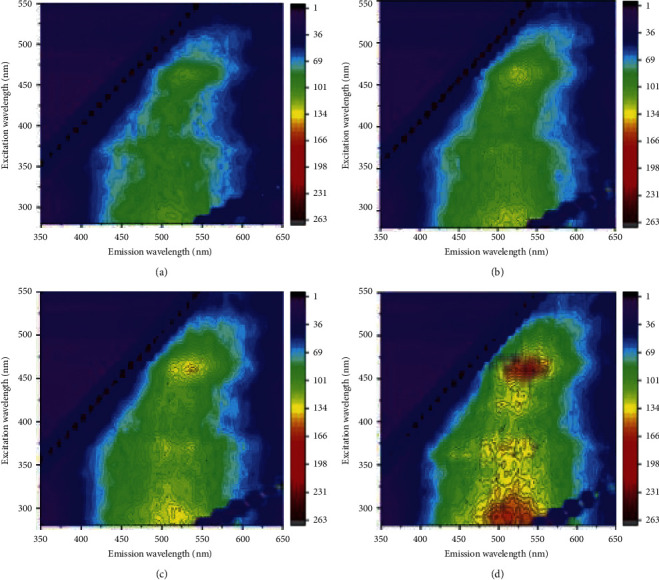
Fluorescence spectra of HA in different maize returning methods on subsoil. *Note.* CK: no straw application; SCR: straw mulching; MBR: straw deep ploughing; SEDI: straw enrichment and deep incorporation. (a) Subsoil: CK. (b) Subsoil: SCR. (c) Subsoil: MBR. (d) Subsoil: SEDI.

The three-dimensional fluorescence spectroscopy of straw returning modes is displayed in Figures 2 and 3. All of the EEM fluorescence spectra of HA exhibited three fluorophores (peaks A–C). The three fluorophores were centered at the Em/Ex wavelength of 430–500/470–550 nm for peak A, 340–370/490–550 nm for peak B, and 270–340/460–550 nm for peak C, which may be related with the amount of humic substances. Among all the treatments, the highest fluorescence intensities of peaks A-C were recorded under SEDI in the 20–40 cm, and the lowest under CK in the 0–20 cm. Generally, higher fluorescence intensity indicates greater proportions of hydroxyl, alkoxyl, and methoxyl [22]. The results showed that SEDI treatment had a slight increase in FI as compared with CK, implying that straw enrichment and deep incorporation were more conducive to improving the straw decomposition and soil HA formation than other treatments (Table 4).

### 3.4. ^13^C NMR Spectra of HA

**Figure 4:**
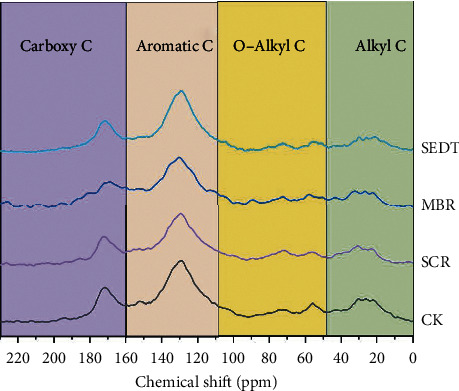
^13^C CPMAS NMR spectra of HA in different maize returning methods. *Note.* CK: no straw application; SCR: straw mulching; MBR: straw deep ploughing; SEDI: straw enrichment and deep incorporation. There were significant differences between different letters representing different treatments (*p* < 0.05).

The ^13^C NMR spectra of HA for each treatment are reported in Figure 4. The spectrum can be divided into 4 main resonance regions, namely, alkyl C (0∼50 ppm), alkoxy C (50∼110 ppm), aromatic C (110∼160 ppm), and carboxyl C (160∼200 ppm) [23]. The peaks at 30 ppm in the alkyl C region were assigned as-CH2-. The peak at 55∼56 ppm in the O-alkyl C region was assigned as methoxyl C in lignin. The peaks at 70∼73 ppm and 104∼105 ppm were carbohydrate C and hydrogen peroxide in polysaccharide, respectively. The peak at 128∼130 ppm in the aromatic C region was ascribed to aryl C. The peak at 171∼173 ppm in the carbonyl C region was indicative of carboxylic acid, amide, and ester [24]. The shape of the spectrum of all treatments of HA shows similar tendencies; however, the absorption peak intensity is significantly different. It indicated that although HA has a similar structure, the HA structure changed after straw returning.

The relative intensity of the HA groups for different treatment is shown in Table 5. Compared with the CK treatment, all the straw returning treatments increased the alkyl C, whereas the O-alkyl C decreased. SCR, MBR, and SEDI treatments were 0.77%, 4.39%, and 16.80% higher than CK treatment in alkyl C and 3.11%, 5.26%, and 8.24% lower in O-alkyl C. These results showed that corn straw return was disadvantageous of accumulation for O-alkyl C; nevertheless, SEDI was beneficial to the formation of alkyl C. Moreover, SEDI treatments decreased aromatic C contents of soil HA by 1.72%. Furthermore, compared with CK, SEDI treatments had no significant difference for carbonyl C; SCR treatment was 2.10% higher than CK treatment. However, MBR treatment decreased by 1.72%. For the ratio of alkyl/O-alkyl and hydrophobic/hydrophilic, all the corn straw return treatments were higher than CK, and SEDI had a higher increasing rate (27.28% and 5.87%). The higher aliphatic C/aromatic C ratio indicated that HA is more aliphatic, and SEDI is more beneficial to improve the straw decomposition and HA formation than the other treatments.

## 4. Discussion

### 4.1. Corn Straw Returning Increased SOC and Humus C Contents

Soil stores most of the ecosystem carbon in the form of organic matter [25], and humus is the most abundant and important component of soil organic matter [7]. Thus, humus is a significant indicator to evaluate soil fertility [26]. Zhang et al. [27] observed that straw returning could increase the content of SOC and humus and improve soil fertility.

After three years, corn straw returning significantly increased SOC, HAC, FA-C, and HM-C contents in both soil depths (Table 2), consistent with previous studies [28–30]. Straw contains carbon, nitrogen, phosphorus, potassium, other nutrients [31] and a certain number of humus fractions [1]. Therefore, its application increases the content of humus C through stem and root exudates [32], which reduces the direct mineralization of soil organic carbon content. Lhadi et al. reported that the degradation of corn straw leads to the formation of humus [33]. The humus C persisted in the soil for a certain time, and then increased the soil carbon sequestration and humus composition content. It led to the increase of the HA/FA ratio, which indicated the humification degree of soil was deepened [3]. Among all the treatments, SEDI was more conductive to increasing SOC, HAC, and FA-C contents in the subsoil (Table 2). Previous studies have shown that the organic carbon content of MBR is higher than that of SCR in the subsoil [34], due to the fact that the corn straw could not be fully mixed with soil under SCR treatment, and decreases its decomposition rate by soil microorganisms [35]. Compared with MBR, the process of deep burying of straw under the SEDI treatment disturbs the subsurface layer of the soil and stimulates microbial activity [36], increasing their metabolic rate [22] and increasing the soil easily oxidizable carbon [37]. The latter processes increase soil carbon sources. Straw returning to the field can promote a significant increase in the carbon content of HA, FA, and HM, while the PQ value increases slightly, but the change is not significant, indicating that the degree of soil maturation and fertility state of the soil transforms in the appropriate direction after straw returning [15].

### 4.2. Corn Straw Returning Decreased the Oxidation Degree and Condensation Degree of HA

The results showed that corn straw returning increased the H/C molar ratio of HA and decreased the O/C molar ratio of HA (Table 3), which indicated that straw returning reduced the condensation degree and oxidation degree of HA structure and simplified the structure of HA. Chen et al. reported that the condensation degree and oxidation degree of HA structure in soil decreased after straw application [5], due to the promotion of microbial activity after straw application, and during the process of microbial metabolic decomposition, HA with complex structure in soil was decomposed, which reduced the stability of HA structure [38]. In addition, the number of newly formed HA oxygen-containing functional groups in the process of straw decomposition was less [39], which reduced the degree of oxidation of HA and made the structure of HA be simpler and younger.

### 4.3. Corn Straw Returning Increased the Hydrophobicity of HA

Fluorescence spectroscopy can be used to measure the structure and properties of compounds and determine the structure of functional groups of humus [40, 41]. Our research has shown that the hydrophobicity of HA structure of the soil was enhanced after the straw returning. In our study, straw application compared with CK increased the intensities of peaks A to C, which indicates that the application of corn straw contains more electron-donating substituents such as hydroxyl, methoxy, and amino [42], and they can increase the fluorescence intensity by increasing the transition probability between the singlet state and the ground state [43]. Gao et al. have shown that straw returning to the field can improve the soil and found that the soil had a higher content of alkoxy carbon and methyl carbon [22, 44], while methyl C was converted from lignin polymerization [45] and was a hydrophobic substance. Therefore, returning straw enhances the hydrophobicity of HA.

Considering the complex peak assignment in the elemental analysis and fluorescence spectroscopy of HA, we evaluated the HA structural characteristics further using the solid-state ^13^C CPMAS NMR technique. Different ways of returning straw to the field led to different changes in the structure of functional groups, mainly manifested as changes in aromatic carbon, alkyl carbon, alkoxy carbon, and carboxyl carbon. Alkyl C (aliphatic compound, methyl C, etc.) is a carbon compound difficult to degrade in soil. Thus, the application of straw results in the increase of alkyl C community and microbial structure [46]. The decomposition of O-alkyl C is mainly due to the fact that cellulose and hemicellulose in plant residues are easily metabolized by microorganisms to utilize organic carbon functional groups. The relative content of alkoxy C slightly increases after straw returning, while during the rapid phase of corn stalk decomposing, the O-alkyl C in the plant residues will be quickly lost into the soil, resulting in a relatively low content of alkoxy C [47]. Aromatic C (mainly from tannin and lignin) is relatively stable. The decrease of the structure in SEDI may be due to the aerobic degradation of lignin by white rot and brown rot fungi through dehydration, demethylation, or cleavage of *β*-O-4 bonds [48]. Carboxyl carbons mainly come from carboxylic acids, amides, and esters [49]. The increase in carboxyl C under SCR treatment might be related to the oxidation of lignin side chains and polysaccharides [39]. The hydrophobicity of HA was determined by the ratio of hydrophobic C (aromatic C and alkyl C) to hydrophilic C (alkoxy C and carboxyl C). The higher the ratio, the stronger the hydrophobicity and the stronger the stability of soil HA [50].

### 4.4. Effects of Different Returning Methods on the Soil HA Structure

Studies have shown that hydrophobicity was meaningful for maintaining the stability of soil organic carbon [50]. Our results showed that straw return enhances the hydrophobicity of HA structure, and SEDI treatment was the most effective (Table 5). Studies have shown that the ratio of high alkyl C and low aromatic C makes the soil organic matter younger [51]. This implies that HA is younger under SEDI treatment. The higher aliphatic C/aromatic C ratio showed (Table 5) that HA was highly aliphatic under SEDI treatment, which made the structure of HA simpler. On the contrary, the aromaticity was enhanced under the MBR and SCR treatment, demonstrating a more complex HA structure. Therefore, straw buried in the subsurface of soil increases the soil oxidizable carbon, stimulates the microbial activity, speeds up the metabolic rate of microorganisms, and promotes the decomposition of HA.

## 5. Conclusion

In this 3-year study, ^13^C NMR spectroscopy and three-dimensional fluorescence spectroscopy were adapted to analyze the structure of soil HA after straw returning. The study concludes the following:

- Adding corn straw into soil significantly increased SOC content and humus composition. Compared to all the rest treatments, SEDI accumulated the largest quantities of SOC and C contents of humic acid, especially in the subsurface soil layer.
- Among the four treatments, SEDI was the most conducive way to enhance the hydrophobicity of the HA structure, which made it more stable, and improve the soil carbon sequestration capacity.

## Figures and Tables

**Table 1:** Basic properties of the tested soil.

Depth (cm)	Organic matter (g/kg)	Total N (mg/kg)	Available P (mg/kg)	Available K (mg/kg)	pH
0∼20	12.20	78.73	95.86	235.80	6.6
20∼40	12.05	76.52	70.17	204.75	6.3

**Table 2:** Effect of different treatments of corn straw on humic substances in black soil.

Depth (cm)	Treatment	HA-C	FA-C	HM-C	PQ (%)
0∼20	CK	3.19 ± 0.10^d^	1.89 ± 0.10^b^	5.31 ± 0.16^c^	62.78 ± 0.01^b^
	SCR	4.24 ± 0.80^a^	2.22 ± 0.08^a^	6.49 ± 0.46^a^	65.67 ± 0.01^a^
	MBR	3.96 ± 0.18^b^	2.20 ± 0.13^a^	6.16 ± 0.12^ab^	64.25 ± 0.02^a^
	SEDI	3.68 ± 0.12^c^	2.08 ± 0.14^a^	5.85 ± 0.25^b^	63.85 ± 0.02^a^
20∼40	CK	3.00 ± 0.16^c^	1.78 ± 0.15^a^	5.05 ± 0.13^c^	62.71 ± 0.03^a^
	SCR	3.22 ± 0.22^bc^	1.86 ± 0.20^a^	5.80 ± 0.19^b^	63.41 ± 0.04^a^
	MBR	3.60 ± 0.10^b^	2.03 ± 0.20^a^	6.01 ± 0.21^b^	63.90 ± 0.04^a^
	SEDI	4.03 ± 0.14^a^	2.13 ± 0.20^a^	6.64 ± 0.38^a^	65.39 ± 0.03^a^

*Note.* CK: no straw application; SCR: straw mulching; MBR: straw deep ploughing; SEDI: straw enrichment and deep incorporation. There were significant differences between different letters representing different treatments (*p* < 0.05).

**Table 3:** Effect of straw returning to the field on HA element composition in soil.

Depth (cm)	Treatment	Element content (g·kg^−1^)				O/C	H/C
		C	H	N	O		
0∼20	CK	487.3 ± 0.8^d^	44.99 ± 0.30^d^	22.71 ± 0.28^d^	477.0 ± 0.8^a^	0.734 ± 0.002^a^	1.108 ± 0.009^c^
	SCR	512.9 ± 0.7^a^	49.54 ± 0.06^a^	27.88 ± 0.10^a^	422.5 ± 0.5^d^	0.618 ± 0.001^d^	1.159 ± 0.001^b^
	MBR	495.1 ± 0.9^b^	47.97 ± 0.10^b^	25.70 ± 0.12^b^	435.2 ± 0.9^c^	0646 ± 0.001^c^	1.140 ± 0.002^a^
	SEDI	491.9 ± 0.8^c^	46.43 ± 0.08c	23.42 ± 0.09^c^	467.5 ± 0.4^b^	0.713 ± 0001^b^	1.133 ± 0.002^a^
20∼40	CK	482.1 ± 0.9^d^	43.21 ± 0.90^d^	21.52 ± 0.10^c^	479.0 ± 0.9^a^	0.745 ± 0.001^a^	1.076 ± 0.024^c^
	SCR	486.8 ± 0.5^c^	44.56 ± 0.08^c^	22.90 ± 0.66^b^	475.0 ± 0.7^b^	0.732 ± 0.002^b^	1.099 ± 0.001^bc^
	MBR	488.7 ± 0.6^b^	45.5 ± 0.17^b^	23.20 ± 0.44^b^	453.1 ± 0.9^c^	0.695 ± 0.001^c^	1.117 ± 0.005^b^
	SEDI	501.2 ± 0.7^a^	48.04 ± 0.16^a^	25.12 ± 0.09^a^	429.2 ± 0.8^d^	0.642 ± 0.001^d^	1.150 ± 0.002^a^

*Note.* CK: no straw application; SCR: straw mulching; MBR: straw deep ploughing; SEDI: straw enrichment and deep incorporation. There were significant differences between different letters representing different treatments (*p* < 0.05).

**Table 4:** Excitation (Ex)/emission (Em) wavelength and fluorescence intensity (FI) of peaks in humic acid after different corn straw return modes.

Soil depth (cm)	Treatment	Peak A		Peak B		Peak C	
		Ex/Em	Intensity (a.u.)	Ex/Em	Intensity (a.u.)	Ex/Em	Intensity (a.u.)
0∼20	CK	460/524	115.02	370/504	105.00	290/503	117.84
	SCR	460/531	154.52	370/523	136.33	290/508	153.54
	MBR	460/523	149.63	370/498	135.81	290/521	141.71
	SEDI	460/528	130.56	370/495	117.72	290/523	129.32
20∼40	CK	460/528	112.86	370/511	112.61	290/515	114.30
	SCR	460/529	125.33	370/493	114.19	290/520	120.14
	MBR	460/537	144.73	370/505.6	126.12	300/522	135.99
	SEDI	460/535	172.34	370/506	143.47	290/509	164.00

*Note.* CK: no straw application; SCR: straw mulching; MBR: straw deep ploughing; SEDI: straw enrichment and deep incorporation.

**Table 5:** Relative intensity (%) of different chemical shift intervals from ^13^C CPMAS NMR spectra of HA examined.

Treatment	Carbonyl C (%) (160–230 ppm)	Aromatic C (%) (110–160 ppm)	O-Alkyl C (%) (50–110 ppm)	Alkyl C (%) (0–50 ppm)	Alkyl C/O-alkyl C	Aliphatic C/aromatic C	Hydrophobic C/hydrophilic C
CK	18.58	53.98	14.45	12.98	0.898	0.508	2.027
SCR	18.97	53.96	14.00	13.08	0.934	0.502	2.034
MBR	18.26	54.49	13.69	13.55	0.99	0.500	2.129
SEDI	18.53	53.05	13.26	15.16	1.143	0.536	2.146

*Note.* CK: no straw application; SCR: straw mulching; MBR: straw deep ploughing; SEDI: straw enrichment and deep incorporation.

## Data Availability

All the data generated or analyzed during this study are available within the article.
